# Attenuating effect of *Polygala tenuifolia* Willd. seed oil on progression of MAFLD

**DOI:** 10.3389/fphar.2023.1253715

**Published:** 2023-10-06

**Authors:** Meiling Xin, Hanlin Wang, Meng Wang, Bendong Yang, Shufei Liang, Xiaoxue Xu, Ling Dong, Tianqi Cai, Yuhong Huang, Qing Wang, Chao Wang, Yuting Cui, Zhengbao Xu, Wenlong Sun, Xinhua Song, Jinyue Sun

**Affiliations:** ^1^ School of Life Sciences and Medicine, Shandong University of Technology, Zibo, Shandong, China; ^2^ College of Life Sciences, Yangtze University, Jingzhou, Hubei, China; ^3^ Key Laboratory of Novel Food Resources Processing, Key Laboratory of Agro-Products Processing Technology of Shandong Province, Ministry of Agriculture and Rural Affairs, Institute of Agro-Food Science and Technology, Shandong Academy of Agricultural Sciences, Jinan, China; ^4^ Shandong Qingyujiangxing Biotechnology Co., Ltd., Zibo, Shandong, China

**Keywords:** metabolic-associated fatty liver disease, *Polygala tenuifolia* Willd., seed oil, SREBPs, NF-κB signaling pathway

## Abstract

**Introduction:** Metabolic-associated fatty liver disease (MAFLD) is a common chronic metabolic disease that seriously threatens human health. The pharmacological activity of unsaturated fatty acid-rich vegetable oil interventions in the treatment of MAFLD has been demonstrated. This study evaluated the pharmacological activity of *Polygala tenuifolia* Willd, which contains high levels of 2-acetyl-1,3-diacyl-sn-glycerols (sn-2-acTAGs).

**Methods:** In this study, a mouse model was established by feeding a high-fat diet (HFD, 31% lard oil diet), and the treatment group was fed a *P. tenuifolia* seed oil (PWSO) treatment diet (17% lard oil and 14% PWSO diet). The pharmacological activity and mechanism of PWSO were investigated by total cho-lesterol (TC) measurement, triglyceride (TG) measurement and histopathological observation, and the sterol regulatory element-binding protein-1 (SREBP1), SREBP2 and NF-κB signaling pathways were evaluated by immunofluorescence and Western blot analyses.

**Results:** PWSO attenuated the increases in plasma TC and TG levels. Furthermore, PWSO reduced the hepatic levels of TC and TG, ameliorating hepatic lipid accumulation. PWSO treatment effectively improves the level of hepatitic inflammation, such as reducing IL-6 levels and TNF-α level.

**Discussion:** PWSO treatment inactivated SREBP1 and SREBP2, which are involved in lipogenesis, to attenuate hepatic lipid accumulation and mitigate the inflammatory response induced via the NF-κB signaling pathway. This study demonstrated that PWSO can be used as a relatively potent dietary supplement to inhibit the occurrence and development of MAFLD.

## 1 Introduction

Metabolic-associated fatty liver disease (MAFLD), also called non-alcoholic fatty liver disease (NAFLD), is a disorder of excessive lipid deposition in hepatocytes caused by factors other than alcohol and other definite factors, and the indicative test result is a hepatic triglyceride (TG) content exceeding 5% after alcohol-free testing (Stefan et al., 2019; Eslam et al., 2020). Many metabolic disorders exist, including hyperglycemia, type 2 diabetes, insulin impedance, dyslipidemia, and adipokine abnormalities, all of which have a significant association with the pathogenesis of MAFLD (Anstee et al., 2013).

MAFLD and its symptoms can be effectively treated with a healthy diet and weight loss, with effects such as a decline in inflammation and improvement in fibrosis (Romero-Gomez et al., 2017). However, many people’s lifestyles and habits do not allow them to maintain an adequate exercise program and healthy diet. As a result, drug intervention is used to ameliorate or prevent MAFLD. Due to the complex mechanism of MAFLD and the fact that Western medicine typically has a single therapeutic target, emphasis on prevention and treatment by traditional Chinese medicine (TCM), which has more complex components, is increasing. Moreover, TCM treatment and prevention methods are widely accepted by the public as safer and more effective than other methods (Gao et al., 2020). TCM with the same origin as medicine and food is characterized by few toxic side effects and high patient compliance. Studies have shown that these TCM practices have had a positive impact on the development of MAFLD, and they are considered an approach for the treatment and prevention of MAFLD (Liu et al., 2014). Numerous researchers have reported that many vegetable and animal oils rich in unsaturated fatty acids might be beneficial for the prevention of lipid metabolism disorders and MAFLD (Henkel et al., 2018; Yang et al., 2019). Recently, vegetable and animal oils rich in 2-acetyl-1,3-diacyl-sn-glycerols (sn-2-acTAGs) were reported, and the high content of sn-2-acTAGs was considered one factor responsible for its pharmacological effects (Smith et al., 2018). However, the effects of oils containing high levels of sn-2-acTAGs on MAFLD were not evaluated.

*Polygala tenuifolia* Willd. is a traditional Chinese medicine that consists mainly of triterpene saponins, sanguinarides and oligosaccharides (Zhao et al., 2020). The main medicinal component of *P. tenuifolia* is the root or the whole herb, which has pharmacological activities such as antidepressant, cholesterol-lowering, anti-inflammatory and anticancer activities (Son et al., 2022). Research has also shown that *P. tenuifolia* and its functional components have a variety of neuroprotective effects, such as the ability to treat and prevent Alzheimer’s disease, neuroinflammatory diseases, and depression (Wang et al., 2017; Yao et al., 2020; Jee et al., 2021). The high level of sn-2-acTAGs in *P. tenuifolia* seed oil (PWSO) might contribute to the pharmacological effects, but the pharmacological activity and potential mechanism of PWSO on MAFLD remain unclear.

In this study, we explored the fatty acid composition of PWSO by gas chromatography (GC) and determined its pharmacological activity on MAFLD in an established mouse model, wherein it inhibited lipid accumulation and liver inflammation by attenuating the activation of the SREBP and NF-κB signaling pathways. We suggest that PWSO may be a potential agent for treating and preventing MAFLD symptoms.

## 2 Materials and methods

### 2.1 Reagents

Seeds of *P. tenuifolia* (Chinese name: Yuan Zhi) were purchased from Xuxin Pharmaceutical Sale (Anhui, China). Commercial kits, including the total cholesterol (TC, A111-1-1), triglyceride (TG, A110-1-1), low-density lipoprotein cholesterol (LDL-C, A113-1-1), high-density lipoprotein cholesterol (HDL-C, A112-1-1), alanine aminotransferase (ALT, C009-2-1), and aspartate aminotransferase (AST, C011-2-1) kits, were purchased from Nanjing Jiancheng Bioengineering Institute (Nanjing, China). The fatty acid standards were purchased from Sigma (United States). Anti-β-actin (K101527P), anti-LAMB1 (K006070P), anti-sterol regulatory element-binding protein 1 (SREBP1, K106528P), anti-fatty acid synthase (FASN, K001685P), anti-acetyl-CoA carboxylase (ACC, 16087-1-AP), anti-3-hydroxy-3-methylglutaryl-CoA reductase (HMGCR, K002888P), anti-IL-6 (K001738P), anti-TNF-α (SEKM-0034), anti-SREBP2 (K106821P), and anti-NF-κB (80979-1-RR) antibodies were purchased from Solarbio (Beijing, China).

### 2.2 Preparation of PWSO

According to previous study (Smith et al., 2018), the dried seeds of *P. tenuifolia* were cold pressed to obtain the crude oil extract (the pressing pressure was 40∼50 MPa, and the pressing temperature was approximately 60°C). Then, the raw *P. tenuifolia* extract was degummed with hot water, deacidified with alkaline solution, and decolorized with white clay for the following experiments. The composition of PWSO has been fully verified in our previous study (Smith et al., 2018), including TLC, GC, MALDI-TOF MS and ^13^C NMR analyses.

### 2.3 Animal experiments

All animal experimental procedures were approved by the Animal Ethics Committee of Shandong University of Technology (the approval date is 17-11-2021, and the approval certification number of the study is YLX20211101). The protocols followed the Guidelines for the Care and Use of Laboratory Animals of Shandong University of Technology. Male Kunming mice (6 weeks old, 18–20 g) were acquired from the Shandong Laboratory Animal Center (Jinan, China) (approval number SCXK 2020-0005) and were housed under standard laboratory conditions with 55%–65% humidity, 25°C ± 3°C, and a 12-h light/dark cycle. All mice were randomly divided into three groups: the normal control group (NC group; *n* = 8), the high-fat diet group (HFD group; *n* = 8), and the PWSO treatment group (PW group; *n* = 8). The mice in the NC group remained on the standard diet throughout the whole experimental period. The mice in the HFD group remained on the HFD throughout the whole experimental period. The PW group was fed a HFD (31% lard oil diet), and after 4 weeks, the mice in the PW group were switched to a PWSO treatment diet (17% lard oil and 14% PWSO diet) for 8 weeks. The whole experiment lasted for 12 weeks. Detailed dietary information can be obtained from Table 1.

**TABLE 1:** Details of the HFD and PWSO diet.

Ingredient	HFD		PWSO diet	
	gm	kcal	gm	kcal
Casein, 80 Mesh	200	800	200	800
L-Cystine	3	12	3	12
Cholesterol	12	0	12	0
PWSO	0	0	111	999
Lard Oil	245	2,205	134	1205.8
Maltodextrin 10	123	500	123	500
Sucrose	68.8	275.2	68.8	275.2
Cellulose, BW200	50	0	50	0
Soybean Oil	25	225	25	225
Mineral Mix, S10026	10	0	10	0
Dicalcium Phosphate	13	0	13	0
Calcium Carbonate	5.5	0	5.5	0
Potassium Citrate, 1 H2O	16.5	0	16.5	0
Vitamin Mix, V10001	10	40	10	40
Vitamin Mix, V10001	2	0	2	0
FD&C Blue Dye	0.05	0	0.05	0
Total	785.85	4,057	785.85	4,057

### 2.4 Biochemical measurements

Body weight and food intake were recorded weekly throughout the experimental period. The mice were sacrificed after 12 weeks of feeding following intraperitoneal injection of pentobarbital sodium (30 mg/kg) for anesthesia. The mice were killed by CO2 inhalation; enucleation was then performed, and blood was collected (0.8–1.2 ml per mouse) and then centrifuged (5 min, 4°C, 3,000 rpm) to obtain plasma. The levels of TC, TG, LDL-C and HDL-C were determined with a microplate reader (Gen 5, BioTek, United States).

### 2.5 Histological observations

Liver tissues from each mouse were collected and fixed with 4% paraformaldehyde for 24 h. Then, the liver tissue specimens were embedded in paraffin and cut into 3-μm-thick sections. Finally, the obtained sections were dyed with hematoxylin and eosin (H&E) and Sirius red for pathological analysis.

### 2.6 Western blot analysis

We evaluated the effects of PWSO on important indicators (such as SREBPs, ACC, FASN, HMGCR and NF-κB) of nuclear and cytoplasmic activity by Western blot analysis. Liver tissue homogenate was obtained by supplementing RIPA lysis buffer with 1% PMSF (Beyotime, China). The protein concentration was measured using a BCA kit (Beyotime, China). Liver tissue samples were separated using 12% SDS‒PAGE and transferred onto PVDF membranes. Then, the membranes were incubated with 5% non-fat milk for approximately 2 h for blocking. Subsequently, the membranes were incubated overnight at 4°C with primary antibodies, including anti-SREBP1 (1:1000, Solarbio, China), anti-ACC (1:1000, Solarbio, China), anti-FASN (1:1000, Solarbio, China), anti-SREBP2 (1:1000, Solarbio, China), anti-HMGCR (1:1000, Solarbio, China), and anti-NF-κB (1:1000, Solarbio, China) antibodies in skim milk powder TBST solution. The membranes were washed three times with TBST and then incubated with the appropriate secondary antibody (1:3500, Solarbio, China) at room temperature for 2 h. The membranes were washed again for development observation. Three samples were selected for analysis.

Protein bands were visualized by using an ECL Plus kit (Beyotime, China), and the band densities were quantified by ImageJ software. β-Actin or LAMB1 was used as a reference, and all protein expression levels were standardized to these band intensities.

### 2.7 Immunofluorescence analysis

We evaluated the effects of PWSO on important indicators (such as SREBP1, IL-6, TNF-α and NF-κB) of nuclear and cytoplasmic activity by immunofluorescence analysis. According to previous study (Yang et al., 2021), the liver slice samples were first fixed with 4% paraformaldehyde for 15 min. The fixed samples were blocked with 2% BSA for 30 min and then incubated with primary antibodies (anti-SREBP1, anti-IL-6, anti-TNF-α and anti-NF-κB, 1:200) overnight at 4°C. Next, the sections were washed and incubated with a FITC-labeled secondary antibody (goat anti-rabbit IgG, 1:500) for 2 h. After the samples were stained with DAPI, a fluorescence microscope was used to acquire images. Three samples were selected for analysis.

### 2.8 Statistical analysis

GraphPad Prism version 8.0.0 was used for graphing. SPSS Statistics V22.0 was used for data analysis. Differences between the groups were analyzed using one-way analysis of variance (ANOVA) and *post hoc* Tukey’s tests. In the *in vitro* and *in vivo* experiments, the data are presented as the means ± standard errors. The significance of differences is denoted as follows: *p < 0.05; **p < 0.01; ***p < 0.001.

## 3 Results

### 3.1 Effects of PWSO on serum lipids in mice fed a HFD

**FIGURE 1:**
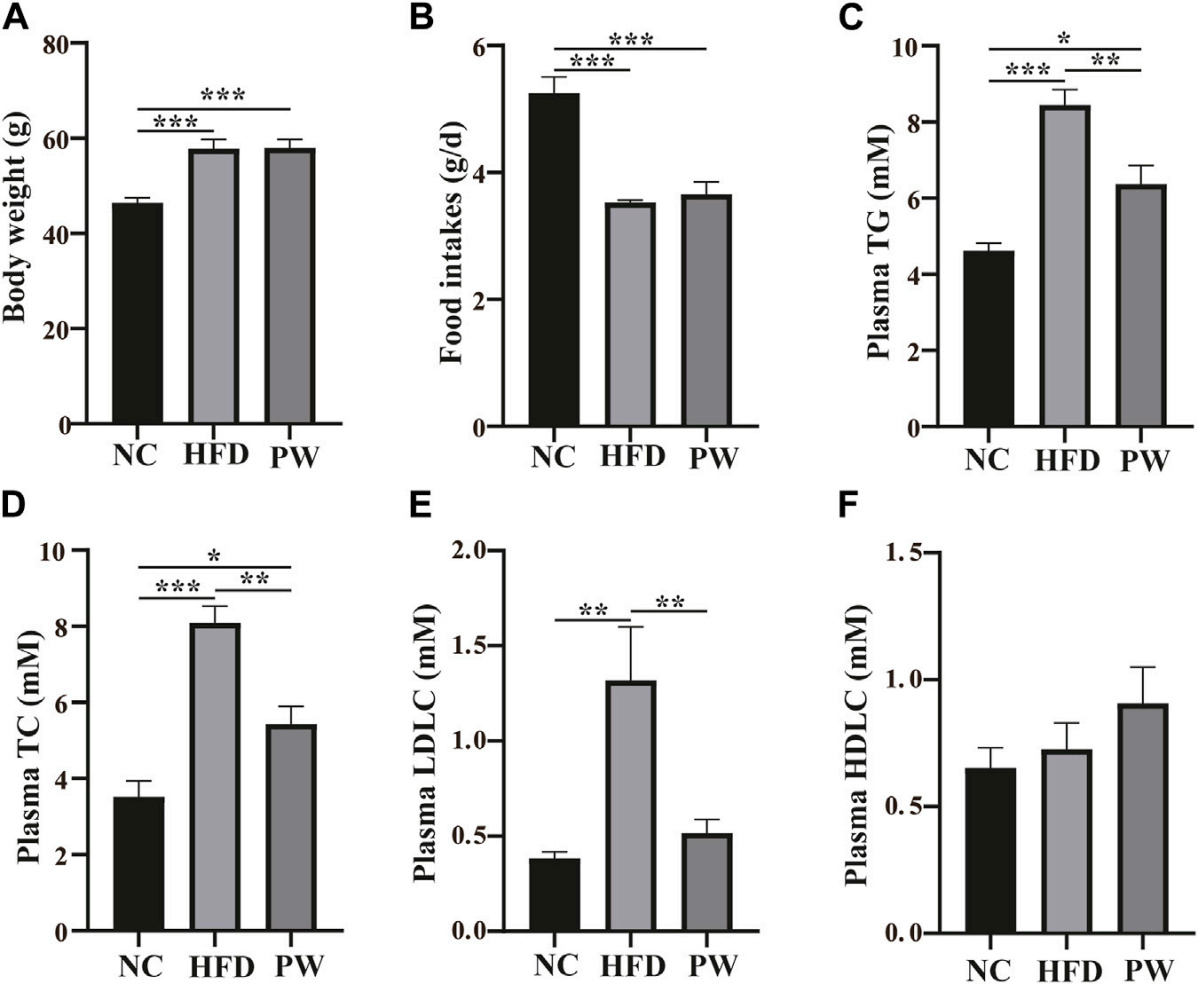
The effect of PWSO on blood lipids in mice with MAFLD. **(A)**: Body weight; **(B)**: Food intake; **(C)** Plasma TG; **(D)**: Plasma TC; **(E)**: Plasma LDL-C; **(F)**: Plasma HDL-C. Statistics: *p < 0.05, **p < 0.01, ***p < 0.001. NC, normal control group; HFD, high-fat diet group; PW, PWSO treatment group. MAFLD, metabolic-associated fatty liver disease; TG, triglyceride; TC, total cholesterol; HDL-C, high-density lipoprotein cholesterol; LDL-C, low-density lipoprotein cholesterol.

After feeding for 12 weeks, relative to the NC group, a clear increase (*p* < 0.001) in body weight was observed in the HFD group (Figure 1A). However, taking the HFD group as the reference, PWSO treatment did not have a significant impact on the weight of mice. Taking the food intake of the NC group as the reference, the food intake of the HFD group decreased (*p* < 0.001) due to the higher energy content in the HFD (Figure 1B). Taking the food intake of the HFD group as the reference, PWSO treatment did not increase food intake. Furthermore, taking the biochemical indicators of the NC group as the reference, there was a significant increasing trend in the plasma levels of TC, TG, and LDL-C (*p* < 0.001, *p* < 0.001, and *p* < 0.01, respectively) in the HFD group (Figures 1C–F). Moreover, PWSO treatment significantly reduced the levels of TC, TG, and LDL-C (*p* < 0.01, *p* < 0.01, and *p* < 0.01, respectively; Figures 1C–E). Regarding the plasma HDL-C level, the NC group, HFD group, and PW group showed consistency (Figure 1F).

### 3.2 Effects of PWSO on serum ALT and AST levels and hepatic lipids in mice fed a HFD

**FIGURE 2:**
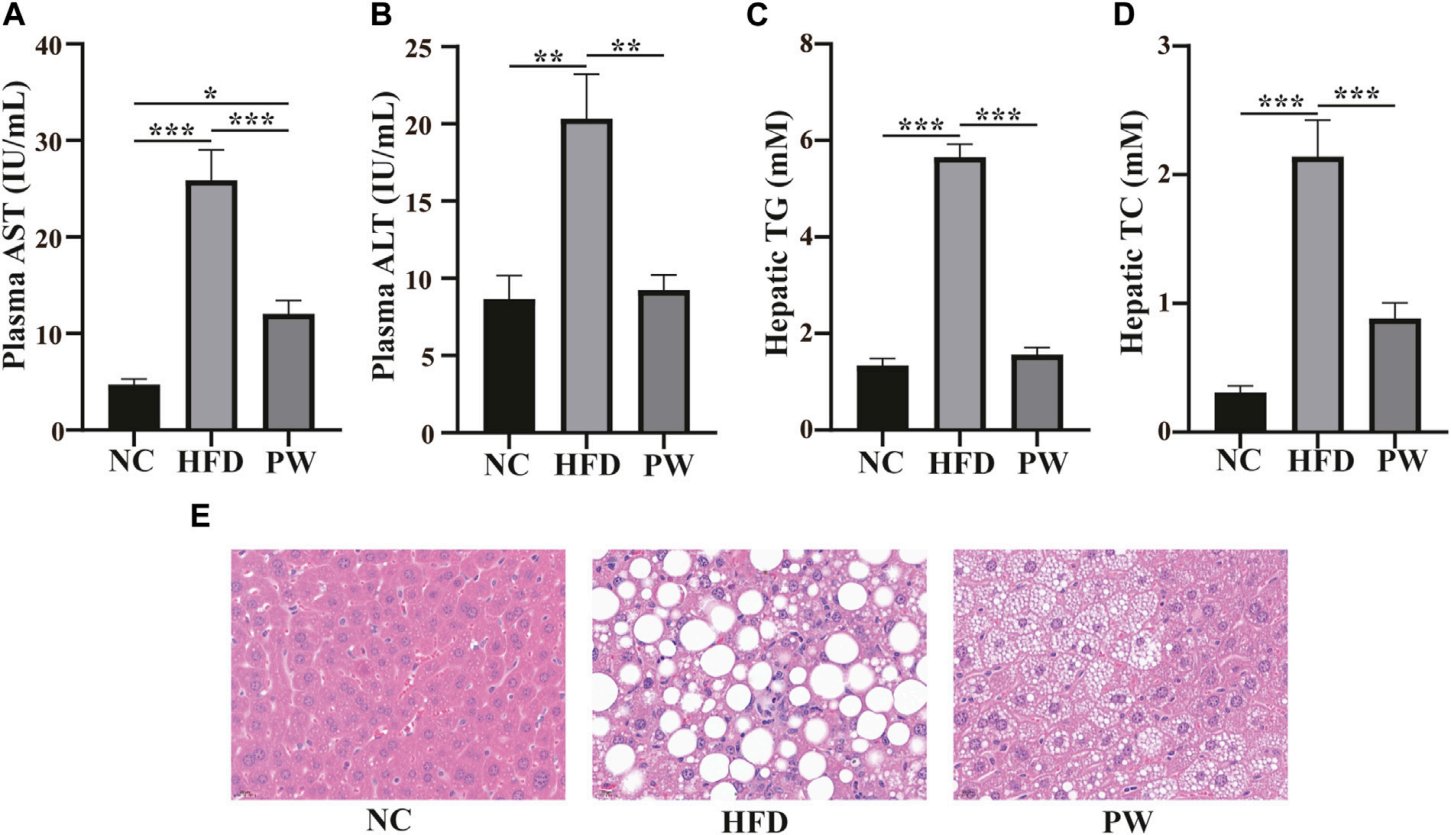
The effect of PWSO on lipids in mice with MAFLD. **(A)**: AST; **(B)**: ALT; **(C)**: Hepatic TG; **(D)**: Hepatic TC; **(E)**: micrographs of hepatic H&E staining; scale bar = 20 µm for H&E staining. Statistics: *p < 0.05, **p < 0.01, ***p < 0.001. NC, normal control group; HFD, high-fat diet group; PW, PWSO treatment group. TC, total cholesterol; TG, triglyceride; ALT, plasma alanine aminotransferase; AST, aspartate aminotransferase; H&E, hematoxylin and eosin.

After feeding for 12 weeks, the levels of serum AST and ALT in the HFD group were significantly higher (*p* < 0.001 and *p* < 0.01, respectively) than those in the NC group. However, a reduction in AST and ALT levels (*p* < 0.001 and *p* < 0.01, respectively; Figures 2A, B) was observed with PWSO treatment. Moreover, the liver TC and TG levels were significantly increased (*p* < 0.001 and *p* < 0.001, respectively) in the HFD group and were significantly decreased in the PW group (*p* < 0.01 and *p* < 0.01, respectively; Figures 2C, D). Robust lipid accumulation in hepatocytes was observed in the HFD group, indicating that HFD-fed mice develop MAFLD (Figure 2E). Moreover, PWSO treatment clearly ameliorated hepatic lipid accumulation. The range of visible lipid droplets was decreased, and the structure of hepatic cells was improved by PWSO treatment (Figure 2E).

### 3.3 Effects of PWSO on SREBP expression and localization and the related signaling pathways in mice fed a HFD

**FIGURE 3:**
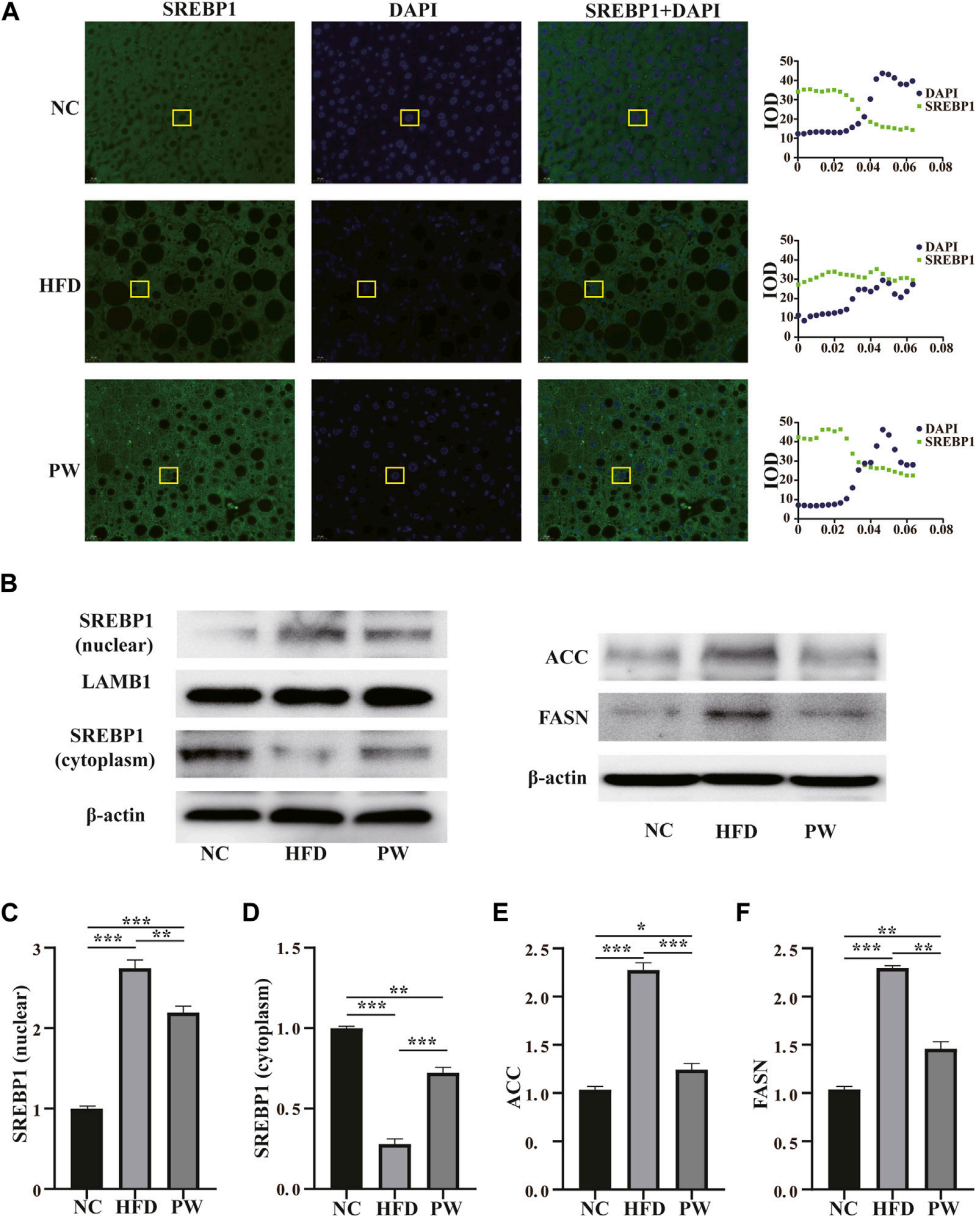
The effect of PWSO on lipogenesis. **(A)**: Immunofluorescence staining for SREBP1 in liver tissue (original magnification, ×200); **(B)**: Expression of SREBP1, ACC and FASN; **(C)**: SREBP1 level in the nucleus; **(D)**: SREBP level in the cytoplasm; **(E)**: ACC level; **(F)**: FASN level. Statistics: *p < 0.05, **p < 0.01, ***p < 0.001. NC, normal control group; HFD, high-fat diet group; PW, PWSO treatment group. FASN, fatty acid synthase; ACC, acetyl-CoA carboxylase; SREBPs, sterol regulatory element-binding proteins.

The immunofluorescence staining results showed that the PW group had increased SREBP1 levels in the cytoplasm (Figure 3A). Moreover, the Western blot analysis showed that the HFD group had a significant increase (*p* < 0.001) in the SREBP1 level in the nucleus when compared to that in the NC group. PWSO treatment significantly decreased (*p* < 0.01) the level of SREBP1 in the nucleus compared with that in the HFD group (Figures 3B, C). However, the HFD group had a significant decrease (*p* < 0.001) in the SREBP1 level in the cytoplasm compared with that in the NC group. Furthermore, PWSO treatment significantly increased (*p* < 0.001) the level of SREBP1 in the cytoplasm (Figures 3B, D). There was a significant increasing trend (*p* < 0.001) in ACC expression observed in the HFD group compared with the NC group, and this trend was reversed (*p* < 0.001) after PWSO treatment (Figures 3B, E). Furthermore, the expression of FASN was significantly increased in the HFD group compared with the NC group (*p* < 0.001). PWSO treatment significantly decreased the expression of FASN (Figures 3B, F).

**FIGURE 4:**
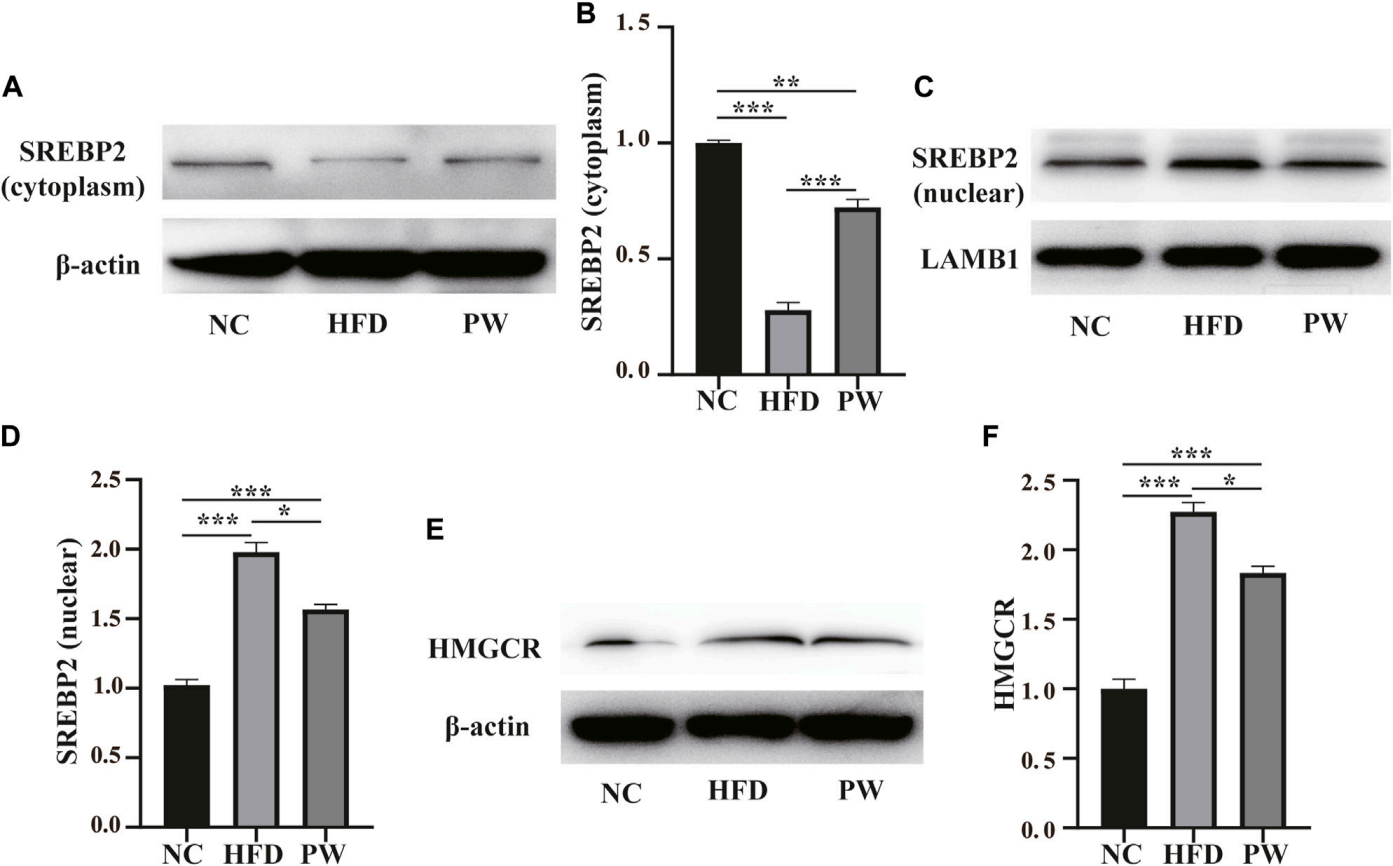
The effect of PWSO on lipogenesis. **(A,B)**: SREBP2 level in the cytoplasm; **(C,D)**: SREBP2 level in the nucleus; **(E,F)**: Expression of HMGCR. Statistics: *p < 0.05, **p < 0.01, ***p < 0.001. NC, normal control group; HFD, high-fat diet group; PW, PWSO treatment group. SREBPs, sterol regulatory element-binding proteins; HMGCR, 3-hydroxy-3-methylglutaryl-CoA reductase.

Western blot analysis showed a clear decreasing trend (*p* < 0.001) in the level of cytoplasmic SREBP2 in the HFD group compared to the NC group. PWSO reversed the HFD-induced decrease in cytoplasmic SREBP2 levels (*p* < 0.001) (Figures 4A, B). Moreover, a clear increasing trend (*p* < 0.001) in the level of nuclear SREBP2 was observed in the HFD group in comparison to the NC group. PWSO significantly prevented the decrease in nuclear SREBP2 levels caused by a HFD (*p* < 0.001) (Figures 4C, D). Western blot analysis showed a clear increasing trend (*p* < 0.001) in the level of HMGCR in the HFD group relative to the NC group. Moreover, in comparison with the HFD group, PWSO treatment significantly reduced the level of HMGCR (Figures 4E, F).

### 3.4 Effects of PWSO on fibrosis and the inflammatory response in mice fed a HFD

**FIGURE 5:**
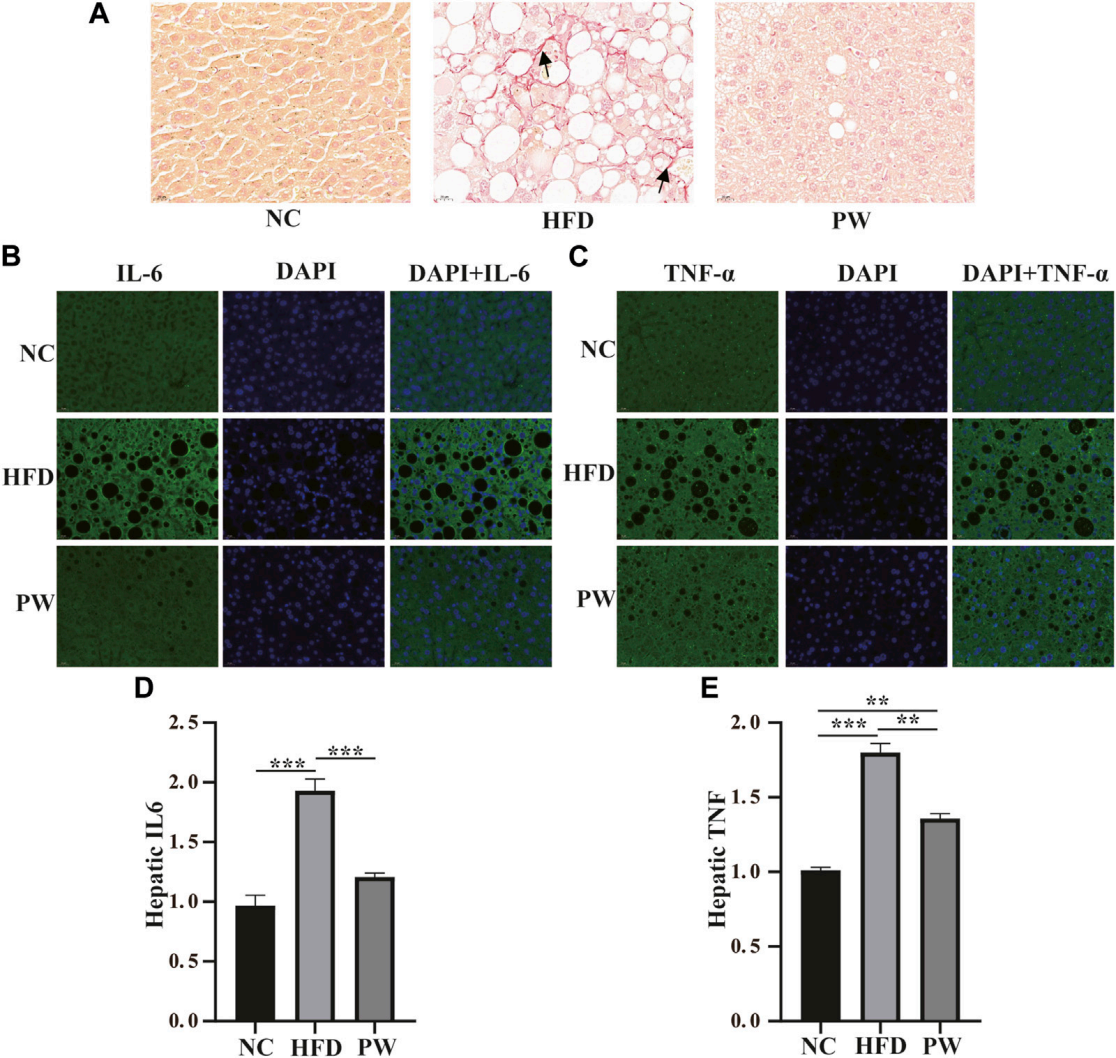
The effect of PWSO on the inflammatory response. **(A)**: Micrographs of hepatic H&E staining; scale bar = 20 µm for H&E staining. **(B)**: Immunofluorescence staining for IL-6 in liver tissue (original magnification, ×200); **(C)**: Immunofluorescence staining for TNF-α in liver tissue (original magnification, ×200); **(D)**: Hepatic IL-6; **(E)**: Hepatic TNF-α. Statistics: *p < 0.05, **p < 0.01, ***p < 0.001. NC, normal control group; HFD, high-fat diet group; PW, PWSO treatment group. H&E, hematoxylin and eosin.

Sirius red staining and immunofluorescence staining showed that the HFD group exhibited severe fibrosis (*p* < 0.001) in comparison to that in the NC group. However, fibrosis was significantly attenuated by PWSO treatment (*p* < 0.001) (Figures 5A–C). Furthermore, after HFD feeding, the PWSO diet and standard diet were administered for 8 weeks, and the IL-6 and TNF-α levels were found to be elevated. Significant increases in hepatic IL-6 and TNF levels (*p* < 0.001 and *p* < 0.001, respectively) were observed in the HFD group compared with the NC group (Figures 5D, E). However, PWSO treatment significantly decreased the levels of IL-6 and TNF (*p* < 0.001 and *p* < 0.01, respectively; Figures 5D, E).

### 3.5 Effects of PWSO on the NF-κB level

**FIGURE 6:**
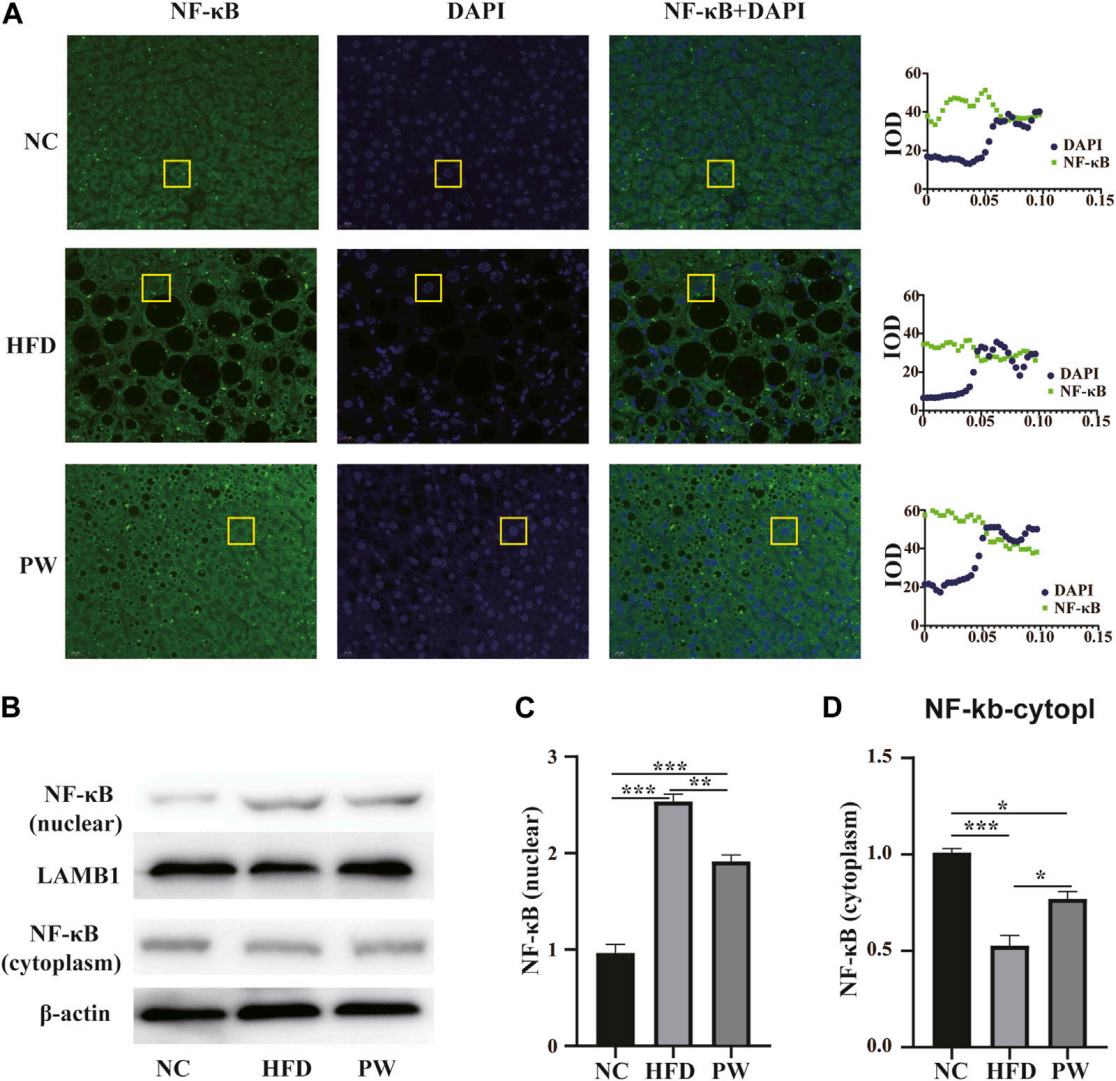
The effect of PWSO on the NF-κB signaling pathway. **(A)**: Immunofluorescence staining for SREBP1 in liver tissue (original magnification, ×200); **(B)**: Expression of NF-κB in the nucleus and cytoplasm; **(C)**: NF-κB level in the nucleus; **(D)**: NF-κB level in the cytoplasm. Statistics: *p < 0.05, **p < 0.01, ***p < 0.001. NC, normal control group; HFD, high-fat diet group; PW, PWSO treatment group.

Immunofluorescence analysis revealed that PWSO increased the NF-κB level in the cytoplasm and decreased the NF-κB level in the nucleus (Figure 6A). In addition, Western blot analysis showed a significant increase (*p* < 0.001) in the level of nuclear NF-κB in the HFD group compared with the NC group (Figure 6B). The nuclear NF-κB level was significantly decreased in the PW group compared with the HFD group (*p* < 0.01) (Figure 6C). However, a significant decrease (*p* < 0.001) in the level of cytoplasmic NF-κB was observed in the HFD group. Moreover, compared to the HFD group, the PW group showed a clear increasing trend (*p* < 0.05) in the level of cytoplasmic NF-κB (Figure 6D).

## 4 Discussion

The pharmacological activity and use of PWSO, which is characterized by high levels of sn-2-acTAGs (Smith et al., 2018), remain to be solved. In our research, the effects of PWSO on MAFLD were examined. Our results showed that treatment with PWSO attenuated the increases in TC and TG levels in MAFLD, thereby ameliorating hepatic lipid accumulation and the inflammatory response. This health benefit was associated with inactivation of SREBPs and inhibition of the NF-κB signaling pathway.

Currently, a multiple hit hypothesis for the pathogenesis of MAFLD is accepted. Genetic susceptibility, epigenetics, hepatic lipid metabolism, insulin sensitivity, lipid peroxidation, and inflammatory responses contribute to the occurrence and development of MAFLD (Friedman et al., 2018; Valenti and Baselli, 2018; Sanyal, 2019; Zhou et al., 2020). Among these contributors, hepatic lipid accumulation is considered the initial cause of MAFLD, and an effective reduction in hepatic lipid accumulation plays an important role in the regression of MAFLD. Inhibiting hepatic lipid synthesis is a main strategy to ameliorate hepatic lipid accumulation. SREBPs are cholesterol sensors in the endoplasmic reticulum that regulate sterol homeostasis through certain feedback mechanisms (DeBose-Boyd and Ye, 2018). Typically, SREBPs exist in a complex in the cytoplasm and are then activated in the Golgi when they receive a biochemical signal (DeBose-Boyd et al., 1999). Active SREBPs enter the nucleus to bind to SREBPs in promoters, which then initiates the expression of genes related to lipogenesis (Athanikar et al., 1998). Therefore, changes in SREBP levels in the cytoplasm and nucleus are important for the activation of the SREBP signaling pathway. The experiments demonstrated that the levels of SREBPs in the nucleus in the PW group were lower than those in the HFD group. Compared with levels in the HFD group, the levels of SREBPs in the cytoplasm were significantly increased in the PW group. It is obvious that PWSO may inhibit the activation of SREBPs and regulate the synthesis of lipogenesis-related enzymes.

In concrete terms, SREBPs are present as three isoforms in mammals: SREBP1c, SREBP1a, and SREBP2 (Brown and Goldstein, 1999). A large amount of SREBP1c is expressed in the liver and mainly regulates the expression of FASN and ACC (Guo et al., 2014; DeBose-Boyd and Ye, 2018). FASN, which is well recognized as a rate-limiting enzyme, plays a vital role in fatty acid synthesis and contributes to the production of TG in the liver. ACC catalyzes the carboxylation of acetyl-CoA to form malonyl-CoA and is an important regulator in the first step of fatty acid synthesis. Our results indicated that PWSO can inactivate SREBP1 and decrease the expression of FASN and ACC, inhibiting the synthesis of TG in the liver (Zhu et al., 2019). Moreover, this result is also the critical cause of the reduction in the plasma TG level. SREBP2 is primarily responsible for sterol metabolism and homeostasis. HMGCR catalyzes the *de novo* synthesis of cholesterol and is directly regulated by SREBP2 (Ren et al., 2017). Its activation has a strong effect on the production of cholesterol. Our results suggested that PWSO can inhibit the activation of SREBP2 and then reduce the expression of HMGCR. Lipid accumulation, a critical event in the development of MAFLD, was decreased by treatment with PWSO.

With hepatic lipid accumulation, lipid peroxidation occurs and initiates inflammation, which leads to cytokine expression and fibrosis in the liver (Welty et al., 2016; Wang et al., 2021). Thus, managing the hepatic inflammatory response is critical for controlling the occurrence and development of MAFLD. NF-κB is responsible for the cellular responses to free radicals and lipid peroxidation (Liu et al., 2017; Poma, 2020). When hepatic cells are damaged by lipid peroxidation, IkB-α is in turn activated and phosphorylated, releasing NF-κB (P65) from the NF-κB/IkB-α complex in the cytoplasm. The p65/RelA dimer undergoes rapid nuclear translocation and binds to NF-κB response elements in target genes via the p65 subunit, thereby initiating the expression of target genes such as TNF-α and IL-6, which eventually leads to tissue inflammation and even causes fibrosis in the liver (Liu et al., 2017). Thus, importantly, the ratio of cytoplasmic to nuclear NF-κB indicates activation of the NF-κB signaling pathway. Our results showed that the concentration of nuclear NF-κB (p65) was higher in the HFD group than in the NC group, and PWSO treatment successfully mitigated the increase in NF-κBp65 levels in the nucleus caused by a HFD. Moreover, the increases in TNF-α and IL-6 expression after activation of the NF-κB signaling pathway were significantly ameliorated by PWSO treatment. These results indicated that PWSO can partially inhibit the inflammatory response in the liver, which efficiently prevents the development of MAFLD.

The high sn-2-acTAG content and unsaturated fatty acid content of PWSO may have beneficial effects compared to other vegetable oils. Unsaturated fatty acids, especially omega-3 and omega-6 unsaturated fatty acids, are generally considered safer than saturated fatty acids (Scorletti and Byrne, 2018). The high content of unsaturated fatty acids in PWSO may also be the main reason for its pharmacological activity. The present study investigated the protective effects of PWSO against MAFLD, but the association between the structure and function of PWSO needs further examination.

It is important to further the use of PWSO as an edible oil. In our study, the mice did not exhibit any discomfort, abnormal mental state, or diarrhea after PWSO administration. These observations provide an important research direction for verifying the safety of PWSO, but further comprehensive toxicological experiments need to be performed.

## 5 Conclusion

PWSO attenuates hepatic lipid accumulation by regulating the activation of SREBPs and the related signaling pathways, inhibits liver inflammation by mitigating the activation of the NF-κB signaling pathway, and ultimately has pharmacological activity in MAFLD. Our study provides not only support for the popularization and use of PWSO but also new perspectives on utilizing the pharmacological activities of plant oils in MAFLD.

## Data Availability

The original contributions presented in the study are included in the article/Supplementary Material, further inquiries can be directed to the corresponding authors.

## Abbreviations

sn-2-acTAGs, 2-acetyl-1,3-diacyl-sn-glycerols; ACC, acetyl-CoA carboxylase; ALT, alanine aminotransferase; AST, aspartate aminotransferase; FASN, fatty acid synthase; GC, gas chromatography; H&E, hematoxylin and eosin; HDL-C, high-density lipoprotein cholesterol; HFD group, high-fat diet group; LDL-C, low-density lipoprotein cholesterol; MAFLD, metabolic-associated fatty liver disease; NAFLD, non-alcoholic fatty liver disease; NC group, normal control group; PWSO, *P. tenuifolia* seed oil; *P. tenuifolia*, Polygala tenuifolia Willd.; PW group, PWSO treatment group; SREBP1, sterol regulatory element-binding protein-1; TC, total cholesterol; TCM, traditional Chinese medicine; TG, triglyceride.
